# The Journal

**Published:** 1853-11

**Authors:** 


The Journal.—Those to whom have been sent the previous numbers of this Journal and have also received this, are informed that they are regarded as *bona fide* subscribers, The P. M. is required by law to notify us when they are refused and also to return the numbers on his hands. A very few have given us notice, and as we have not heard from others we are left to infer that they desire a continuance *of course.*

We have received from a number of our medical brethren the most flattering commendations of the Journal, and our exchanges heve welcomed our "*western stranger*" into the ranks of the medical periodicals of the day with the warmest cordiality, and express the kindest hopes and prayers for its success, Not only this, but without a singly exception they have spoken well of the "matter and manner."

Our subscription list is regularly increasing, and from present prospects it will show at the end of the present volume a large subscription list. All are convinced that it is destined to continue the organ of the profession of the State, and therefore they do not hesitate to order it. From one town alone, whose population we do not know but presume it to be quite small, three physicians ordered it by the same mail, enclosing payment for the current year. Such liberality and magnanimity are worthy the members of our exalted profession.

